# Effects of STAT3 on aging-dependent neovascularization impairment following limb ischemia: from bedside to bench

**DOI:** 10.18632/aging.204122

**Published:** 2022-06-13

**Authors:** Wei-Ting Chang, You-Cheng Lin, Chon-Seng Hong, Po-Sen Huang, Yu-Wen Lin, Zhih-Cherng Chen, Tsung-Hsien Lin, Ting-Hsing Chao

**Affiliations:** 1 Division of Cardiology, Department of Internal Medicine, Chi-Mei Medical Center, Tainan, Taiwan; 2 Department of Biotechnology, Southern Taiwan University of Science and Technology, Tainan, Taiwan; 3 Institute of Clinical Medicine, College of Medicine, National Cheng Kung University, Tainan, Taiwan; 4 Division of Plastic and Reconstructive Surgery, Department of Surgery, Chi-Mei Medical Center, Tainan, Taiwan; 5 Faculty of Medicine, College of Medicine, Kaohsiung Medical University, Kaohsiung, Taiwan; 6 Division of Cardiology, Department of Internal Medicine, Kaohsiung Medical University Hospital, Kaohsiung, Taiwan; 7 Division of Cardiology, Department of Internal Medicine, National Cheng Kung University Hospital, College of Medicine, National Cheng Kung University, Tainan, Taiwan

**Keywords:** aging, limb ischemia, STAT3, MALE, angiogenesis

## Abstract

Aging is a major risk factor for ischemic hypoxia-related diseases, including peripheral artery diseases (PADs). Signal transducer and activator of transcription 3 (STAT3) is a critical transcription activator in angiogenesis. Nevertheless, the effect of aging on endothelial cells and their responses to hypoxia are not well studied. Using a hindlimb hypoxic/ischemic model of aged mice, we found that aged mice (80-100-week-old) expressed significantly lower levels of angiogenesis than young mice (10-week-old). In our *in vitro* study, aged endothelial cells (≥30 passage) showed a significant accumulation of *β*-galactosidase and a high expression of aging-associated genes, including p16, p21, and hTERT compared with young cells (<10 passage). After 24 hours of hypoxia exposure, proliferation, migration and tube formation were significantly impaired in aged cells compared with young cells. Notably, STAT3 and angiogenesis-associated proteins such as PI3K/AKT were significantly downregulated in aged mouse limb tissues and aged cells. Further, using STAT3 siRNA, we found that suppressing STAT3 expression in endothelial cells impaired proliferation, migration and tube formation under hypoxia. Correspondingly, in patients with limb ischemia we also observed a higher expression of circulating STAT3, associated with a lower rate of major adverse limb events (MALEs). Collectively, STAT3 could be a biomarker reflecting the development of MALE in patients and also a regulator of age-dependent angiogenesis post limb ischemia. Additional studies are required to elucidate the clinical applications of STAT3.

## INTRODUCTION

With an increasing prevalence, peripheral arterial disease (PAD) has become a new threat to public health [1]. It involves aberrant vascular endothelial cell proliferation and angiogenesis [2, 3]. Patients with PAD may develop major adverse limb events (MALEs) during the late stage of disease propagation that markedly reduce blood flow to the extremities and then progress to severe resting pain and even tissue loss [1, 2]. Although surgical or endovascular revascularization has been used to treat PAD, some patients fail to tolerate these intricate and high-risk surgeries [4].

Treatment for PAD remains a formidable challenge to clinicians [2, 4]. Among the strategies for limb salvage, angiogenesis, in which vascular cells proliferate and form new collateral vessels, is an optimal option [2]. Through angiogenesis and vascular remodeling, endothelial cells are the major responders to ischemic hypoxia [2, 5]. Nevertheless, only some patients successfully develop angiogenesis during the process of limb ischemia. The determining factor in angiogenesis remains uncertain. Signal transducer and activator of transcription 3 (STAT3) has been found to be a critical transcription activator in angiogenesis and apoptosis [6, 7]. The relationship between the STAT3 signaling pathway and angiogenesis has been examined in carcinogenic processes, but few studies have reported on the role of this molecule in atherosclerotic plaques [8, 9]. However, the effect of aging on endothelial cells and their responses to hypoxia are not well studied. In this translational study, we aimed to investigate the role of STAT3 in angiogenesis in patients with PAD and its impact on clinical outcomes.

## RESULTS

### Impaired angiogenesis of aged mice post hindlimb ischemia surgery

**Figure 1:**
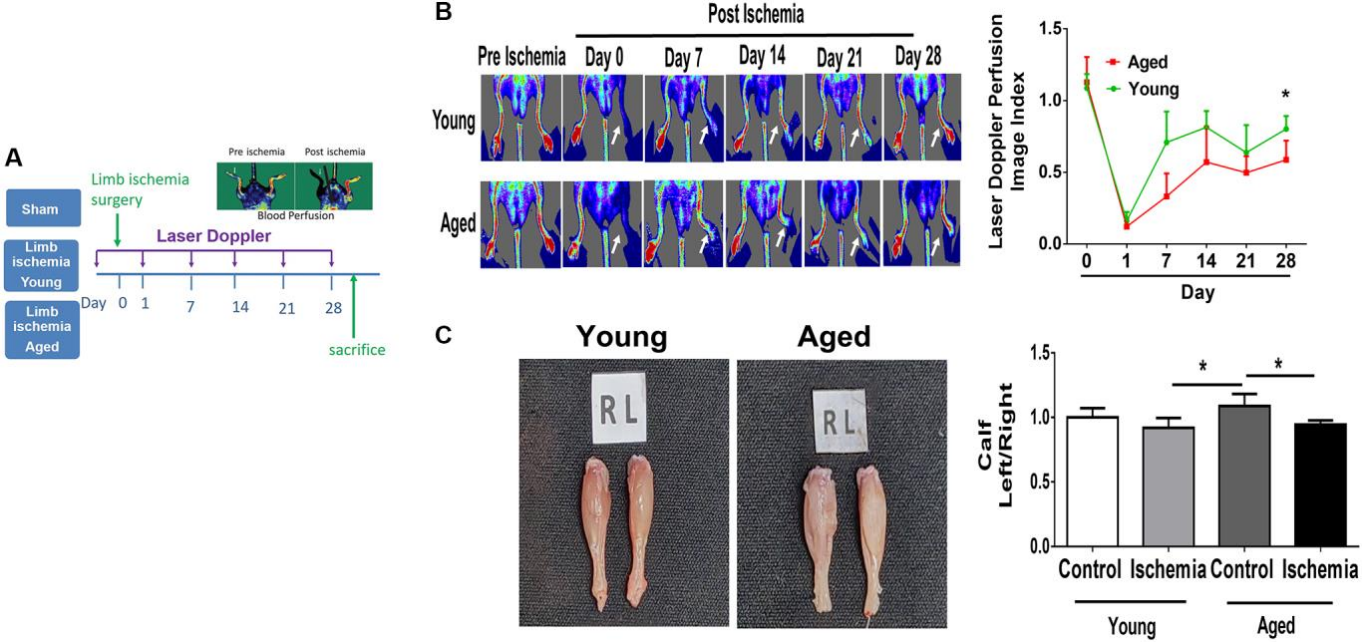
**Impaired angiogenesis of aged mice post hindlimb ischemia surgery.** (**A**) The study design of limb ischemia in young (10 w/o) and aged (80–100 w/o) mice. (**B**) Representative images of laser Doppler perfusion flow in limb ischemia in young and aged mice. (**C**) Representative images and weight quantifications of harvested ischemic (left) limbs compared with nonischemic (right) limbs. *N* = 6, ^*^P < 0.05.

**Figure 2:**
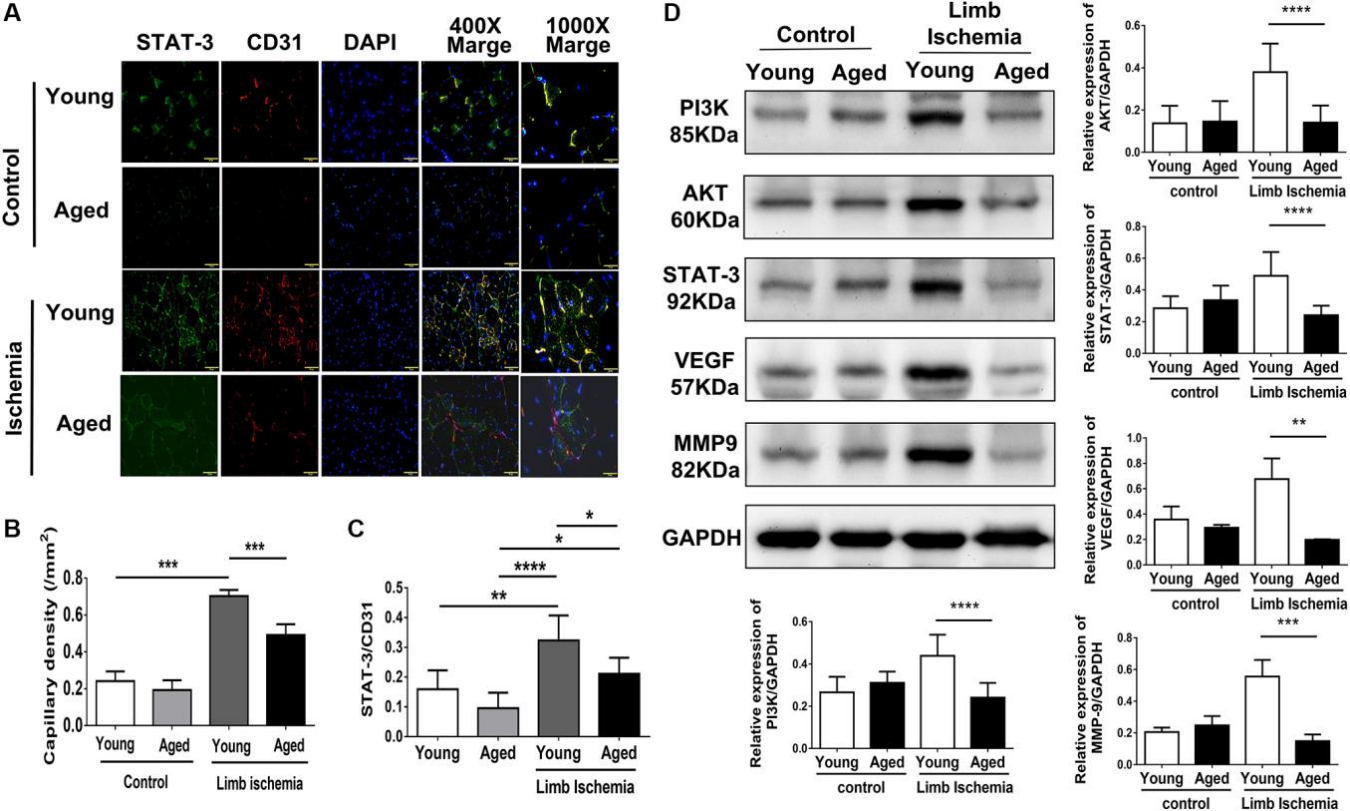
**Impaired capillary density, expression of STAT3 and proliferation-associated proteins in young and aged mice post hindlimb ischemia surgery.** (**A**) Representative images and quantification of CD31 immunostaining representing capillary density (red; left panel) and STAT3 (green) expressions in endothelial cells (red; CD 31 positive; right panel). Cell nuclei were stained with DAPI (blue); (**B**) The quantification of capillary density and (**C**) STAT3 expression; (**D**) Representative blots and quantification of PI3K, AKT, STAT3, VEGF, and MMP9 protein expression in the ischemic limbs of young and aged mice. The experiment was repeated in triplicate, ^*^P < 0.05, ^**^P < 0.01, ^**^P < 0.001 and ^****^P < 0.0001.

Using laser Doppler imaging (LDI), we found that compared with young mice (10-week-old), aged mice (80-100-week-old) presented with impaired angiogenesis after hindlimb ischemia surgery (Figure 1A, 1B). Additionally, at the end of the experiment, the weights of the harvested limbs were measured. In young mice, the muscle weight of the ischemic limb was only slightly reduced, but in aged mice, there was a significant reduction in the weight of the ischemic limb compared with the nonischemic limb (Figure 1C). Following 28 days of hindlimb ischemic surgery, in young mice, hindlimb ischemia surgery triggered a significant increase in capillary density, as shown by staining with CD31, a marker specific for capillaries, but in aged mice, the augmented capillary density was attenuated (Figure 2A, 2B). Additionally, we found that expressions of STAT3 were increased on CD31 positive cell of young mice but decreased on that of aged mice (Figure 2A, 2C). The expression of STAT3 and proliferation-associated proteins, including PI3K, AKT, VEGF and MMP9 [5, 6, 9, 10], was measured in the hindlimb muscles by western blot. Interestingly, we found that limb ischemic surgery activated the expression of STAT3 and the abovementioned markers for proliferation in young mice but not in aged mice (Figure 2D). In order to confirm the expression of MMP9 and VEGF in endothelial cells, the endothelial cells were double stained with CD31 and MMP9 or CD31 and VEGF. Both in young and aged mice, immunofluorescence studies revealed that the expression of MMP9 and VEFGF in CD31 expressing endothelial cells (Supplementary Figure 1A, 1B). After ischemic surgery, hindlimb ischemia surgery triggered a significant increase MMP9 and VEGF expression in endothelial cell of young mice. Compared to young mice, the MMP9 and VEGF expression were significantly decreased in endothelial cell of aged mice after hindlimb ischemia surgery.

### Aging attenuates proliferation, migration and tube formation in HUVECs

**Figure 3:**
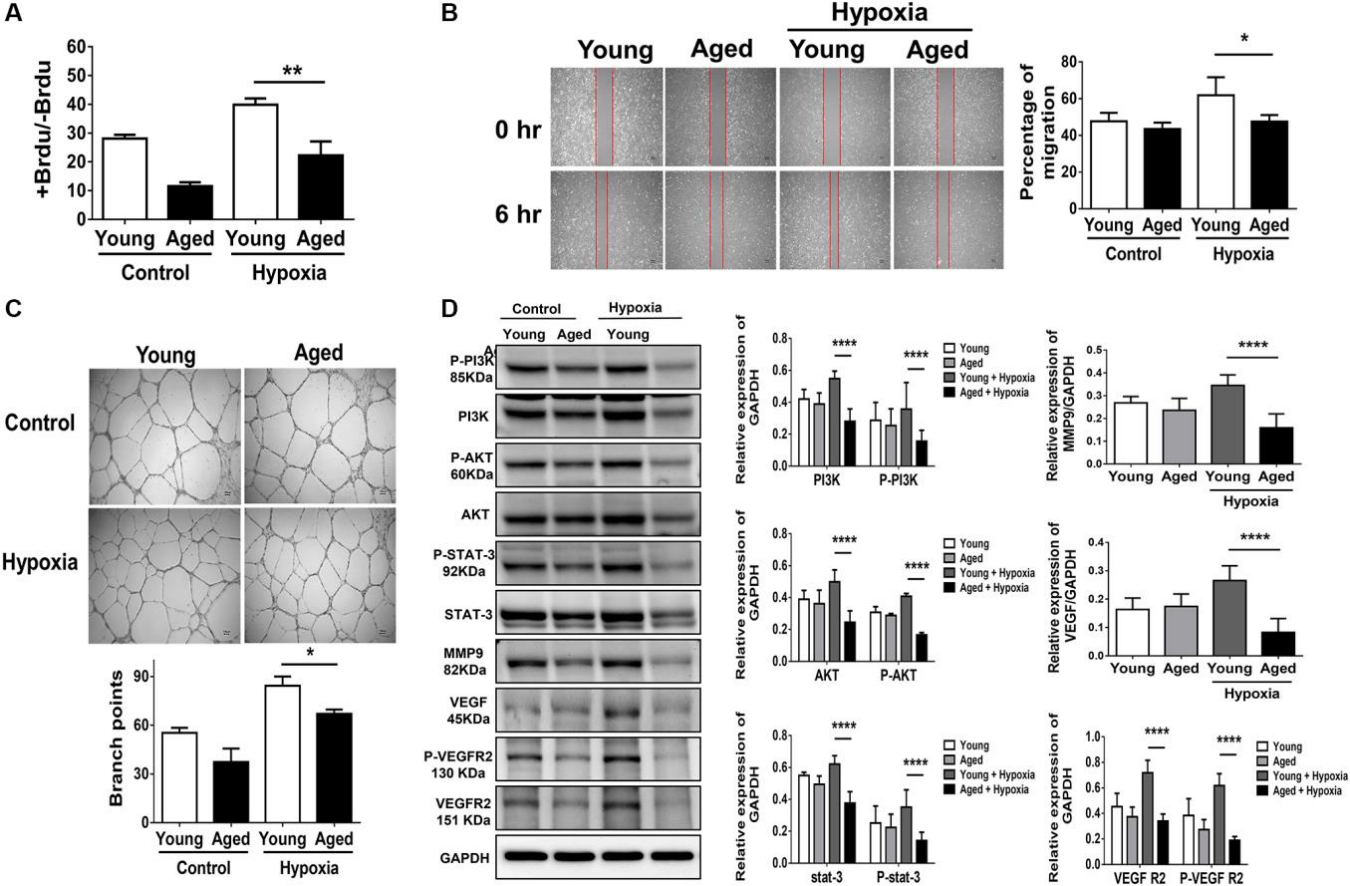
**Aging attenuates proliferation, migration and tube formation in human umbilical vein endothelial cells (HUVECs).** (**A**) Cell proliferation, evaluated by measuring BrdU incorporation into cells, (**B**) cell migration after 24 hours, (**C**) tube formation, evaluated by the branched points, and (**D**) expressions of P-PI3K, PI3K, P-AKT, AKT, P-STAT3, STAT3 and proliferation-associated proteins of MMP9, VEGF, VEGFR2 and the phosphorylation form of VEGFR2 in young (<10 passage) and aged HUVECs (≥30 passage) with and without hypoxia treatment for 24 hours. The experiment was conducted in triplicate, ^*^P < 0.05, ^**^P < 0.01, ^***^P < 0.001 and, ^****^P < 0.0001.

To establish a cellular model mimicking aged capillaries, HUVECs sub-cultured to achieve more than 30 passages were defined as aged cells compared with those within ten passages, defined as young cells [11]. To provide evidence regarding the aging process in these cells, β-galactosidase, an extensively utilized biomarker for the detection of cellular senescence, and p16, p21, and hTERT genes involved in DNA damage and senescence were measured [11]. The aged cells showed a significant accumulation of β-galactosidase and high expression of aging-associated genes (Supplementary Figure 2). After hypoxia stimulation, proliferation, migration and tube formation were, at least in part, augmented in young HUVECs. In contrast, hypoxia-triggered proliferation, migration and tube formation were significantly attenuated in aged HUVECs (Figure 3A–3C). Notably, compared with significant increases in the protein expression of P-STAT3 and the proliferation-associated proteins (e.g., P-PI3K and P-AKT) as well as angiogenesis-associated proteins including VEGF, VEGF receptor 2 (VEGFR2), P- VEGFR2 and MMP9 in young HUVECs post hypoxia, hypoxia-activated protein expression was significantly reduced in aged HUVECs (Figure 3D).

### STAT3 mediates hypoxia-induced proliferation, migration and tube formation in HUVECs

**Figure 4:**
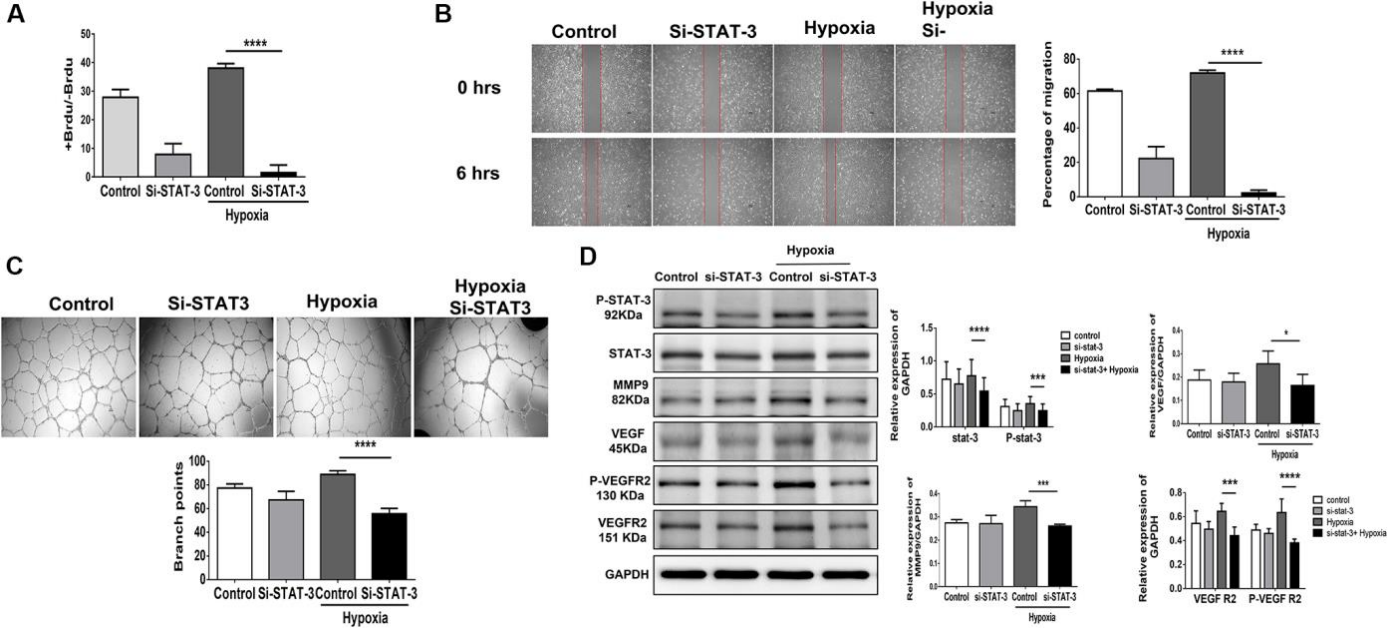
**Knocking down STAT3 suppresses proliferation, migration and tube formation in human umbilical vein endothelial cells (HUVECs).** (**A**) Cell proliferation, evaluated by measuring BrdU incorporation into cells, (**B**) cell migration after 24 hours, (**C**) tube formation, evaluated by the branched points, and (**D**) phosphorylation protein expression of P-STAT3 and proliferation-associated proteins, including MMP9, VEGF, VEGFR2 and the phosphorylation form of VEGFR2, in HUVECs treated with scramble or STAT3 small interfering RNA (siSTAT3) under normoxia or hypoxia for 24 hours. The experiment was repeated in triplicate, ^*^P < 0.05, ^***^P < 0.001, and ^****^P < 0.0001.

Using STAT3 siRNA (10 nM for 48 hours), we suppressed half of the STAT3 mRNA expression in HUVECs (Supplementary Figure 3). We found that suppressing STAT3 expression in HUVECs impaired proliferation, migration and tube formation under hypoxia (Figure 4A–4C). Additionally, the hypoxia-triggered expression of angiogenesis-associated proteins, including VEGF, VEGFR2, P-VEGFR2 and MMP9 [5, 9, 10], was significantly decreased in HUVECs treated with STAT3 siRNA (Figure 4D). This implies that compared with aging individuals, young individuals with endothelial high expression of STAT3 may facilitate the proliferation, function and angiogenesis of endothelial cells in the microenvironment of limb ischemia. Additionally, using PI3K siRNA (10 nM for 48 hours), the PI3K expression in HUVECs was significant suppressed (Supplementary Figure 4A). The cell proliferation, migration, and tube formation under hypoxia were impaired while suppressing PI3K expression in HUVECs (Supplementary Figure 4B–4D). Taken together, either suppressing STAT3 or PI3K expression reduces the capabilities of proliferation, migration, and tube formation in HUVECs.

### Old age with low circulating STAT3 was associated with more MALE

**Figure 5:**
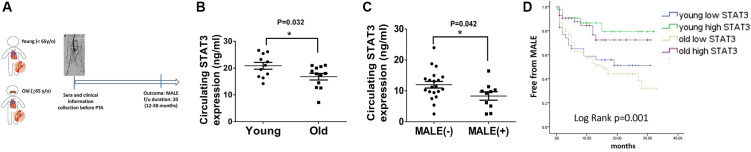
**Lower circulating STAT3 expression in elderly individuals is associated with more major adverse limb events (MALEs).** (**A**) The design of a clinical study focusing on patients with peripheral artery disease (PAD). (**B**) The expression of circulating STAT3 in young (<65 y/o) and old (≥65 y/o) patients. (**C**) The expression of circulating STAT3 in patients with or without MALEs. (**D**) A Kaplan–Meier plot of MALEs among young patients with high STAT3, young patients with low STAT3, old patients with high STAT3, and old patients with high STAT3 (*N* = 11–20, ^*^P < 0.05), ^*^The cutoff value of STAT3 is defined as 15 ng/ml.

To extend the application of STAT3 in a clinical aspect, we prospectively collected the sera and clinical information in patients with PAD preparing for peripheral interventions. After excluding 14 patients who failed to meet the inclusion criteria, this study prospectively enrolled 216 patients receiving PTA for PAD (Figure 5A). The patients’ average age was 68.4 ± 12.6 years (Table 1), while 81 and 135 of them were defined as young (<65 y/o) and old (≥65 y/o), respectively. Compared with young patients, old patients were prone to have a lower body mass index (BMI), a higher systolic blood pressure and worse renal function. Notably, the levels of circulating STAT3 were significantly lower in old patients than in young patients with PAD (13.82 ± 6.2 ng/ml vs. 17.7 ± 6.6 ng/ml, *p* = 0.001) (Figure 5B). During the follow-up period, more old patients reached the endpoint of MALEs than young patients (39.2% vs. 25.9%, *p* = 0.04). Alternatively, when dividing patients according to the development of MALEs (Supplementary Table 1), we found that patients with MALEs were prone to be older (70.1 ± 11.5 y/o vs. 62.5 ± 18.1 y/o, *p* = 0.05) with more coronary artery disease (CAD), heart failure (HF), chronic kidney disease (CKD) or previous stroke than those without MALEs. Likewise, patients who developed MALEs had lower circulating STAT3 levels than those free from MALEs (11.1 ± 5.9 ng/ml vs. 17.2 ± 6.2 ng/ml, *p* = 0.001) (Figure 5C). In Cox regression analysis, we found that a history of HF (HR: 2.11, CI: 1.18–3.78, *p* = 0.012), CKD (HR: 1.95, CI: 1.18–3.2, *p* = 0.008) and the expression of circulating STAT3 (HR: 0.9, CI: 0.86–0.94, *p* = 0.001) were significantly associated with the occurrence of MALEs (Table 2). Among those risk factors, only CKD (HR: 1.82, CI: 1.11–3.01, *p* = 0.018) and circulating STAT3 (HR: 0.91, CI: 0.87–0.94, *p* = 0.001) remained significantly correlated with MALEs in the multivariable analysis. Further, using the cutoff value of 15 ng/ml, circulating STAT3 could be a sensitive biomarker to predict the development of MALEs in patients with PAD (HR: 0.35, CI: 0.19–0.63, *p* = 0.001). When dividing patients according to age and circulating STAT3, those with low expression of circulating STAT3 (<15 ng/ml) had a lower chance of being free from MALEs (Figure 5D). Among them, old patients with low STAT3 presented with the worst outcomes of MALEs compared with the other groups.

**Table 1:** The baseline characteristics of patients with peripheral arterial disease, divided by ages (PAD) (*N* = 216).

**Parameters**	**Young (<65 y/o) (*N* = 81)**	**Old (≧65 y/o) (*N* = 135)**	***P* value**
Age (y/o)	55.4 ± 8.3	76.2 ± 7.2	0.001
Male gender, *n* (%)	66 (81.4)	73 (54.1)	0.001
BMI (kg/m^2^)	26.1 ± 5	23.73 ± 3.9	0.001
Heart rate (bpm)	83.2 ± 16.5	80.4 ± 17.3	0.036
SBP (mmHg)	147.6 ± 29.7	156.2 ± 28.5	0.015
DBP (mmHg)	80.6 ± 14.5	75.3 ± 15.9	0.230
Smoking, *n* (%)	67 (82.71)	70 (51.85)	0.001
Diabetes mellitus, *n* (%)	46 (56.79)	84 (62.22)	0.47
Hypertension, *n* (%)	42 (51.85)	106 (78.51)	0.001
CAD, *n* (%)	38 (46.91)	58 (42.96)	0.45
Heart failure, *n* (%)	7 (8.641)	20 (14.81)	0.2
Hyperlipidemia, *n* (%)	37 (45.67)	51 (37.77)	0.25
Previous stroke, *n* (%)	9 (11.11)	22 (16.2)	0.32
Cancer, *n* (%)	4 (4.93)	12 (8.88)	0.42
CKD (including H/D), *n* (%)	15 (18.5)	35 (25.9)	0.05
eGFR (ml/min/1.73m^2^)	66.61 ± 35.9	52.48 ± 30.2	0.004
ALT(mg/dl)	25.1 ± 16.5	25.65 ± 32.8	0.906
Total Cholesterol(mg/dl)	162.1 ± 44.8	148.14 ± 44.8	0.119
LDL(mg/dl)	97.6 ± 38.2	86.32 ± 46.3	0.215
Triglyceride (mg/dl)	204.8 ± 24.9	156.34 ± 38.8	0.312
Circulating STAT3 (ng/ml)	17.7 ± 6.6	13.82 ± 6.2	0.001
MALE, *n* (%)	21 (25.9)	53 (39.2)	0.04

Data are *n* (%) or mean ± standard error; *P <* 0.05 as significance. Abbreviations: BMI: body mass index; SBP: systolic blood pressure; DBP: diastolic blood pressure; CAD: coronary artery disease; CKD: chronic kidney disease; eGFR: estimated Glomerular filtration rate; ALT: Alanine aminotransferase; LDL: low-density lipoprotein; STAT3: Signal transducer and activator of transcription 3; MALE: major adverse limb events.

**Table 2:** The univariate and multivariable analysis of MALE in patients with peripheral arterial disease (PAD).

	**Univariate**		**Multivariable**			
	**HR (95% CI)**	***P***	**Model 1**		**Model 2**	
			**HR (95% CI)**	***P***	**HR (95% CI)**	***P***
Old age (≧65 y/o)	1.56 (0.94–2.6)	0.08	1.74 (0.96–3.13)	0.064	1.73 (0.96–3.13)	0.067
Male	1.06 (0.66–1.7)	0.8				
Previous stroke	0.33 (0.13–0.82)	0.017				
BMI	1.008 (0.95–1.06)	0.77				
Hypertension	0.79 (0.49–1.28)	0.35				
SBP	1.003 (0.99–1.01)	0.42				
CAD	1.36 (0.86–2.15)	0.186				
Heart failure	2.11 (1.18–3.78)	0.012	1.17 (0.69–1.97)	0.549	1.29 (0.77–2.17)	0.321
CKD (stage≧3 including ESRD)	1.95 (1.18–3.2)	0.008	1.82 (1.11–3.01)	0.018	1.89 (1.15–3.11)	0.012
Circulating STAT3	0.9 (0.86–0.94)	0.001	0.91 (0.87–0.94)	0.001		
Circulating STAT3≧15 ng/ml	0.31 (0.17–0.56)	0.001			0.35(0.19–0.63)	0.001

Abbreviations: BMI: body mass index; SBP: systolic blood pressure; DBP: diastolic blood pressure; CAD: coronary artery disease; CKD: chronic kidney disease; eGFR: estimated Glomerular filtration rate; ALT: Alanine aminotransferase; LDL: low-density lipoprotein; STAT3: Signal transducer and activator of transcription 3; MALE: major adverse limb events. Model 1 including parameters of old age, CKD, HF, STAT3 during follow-up while STAT3 as continuous variables; Model 2 including parameters of old age, CKD, Heart failure, STAT3≧15 ng/ml during follow-up while all parameters as categorical variables.

## DISCUSSION

In this study, we found that among patients with PAD, generally, old patients expressed a lower level of circulating STAT3 than younger patients. Notably, low STAT3 expression was positively correlated with the subsequent development of MALEs, including limb loss or repeated interventions. Likewise, the aged mice expressed significantly lower angiogenesis and tissue-specific STAT3 expression than the young mice 28 days post hindlimb hypoxic/ischemic surgery. After 24 hours of hypoxia, along with low STAT3 expression, proliferation, migration and tube formation were significantly impaired in aged HUVECs compared with young HUVECs. Furthermore, we found that suppressing STAT3 expression attenuated the proliferation, migration and tube formation capabilities of HUVECs under hypoxia. A summary of our findings is illustrated in Graphic abstract.

**Figure:**
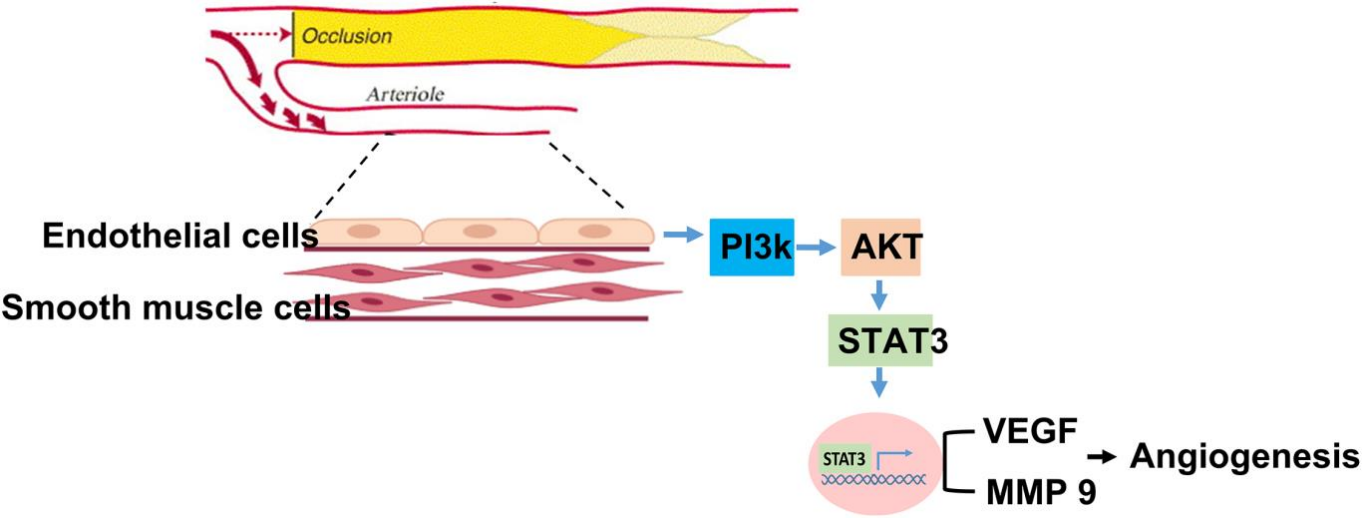
**Graphic abstract.** Summary of STAT3 in regulating aging association attenuation of angiogenesis in limb ischemia.

With its increasing prevalence, PAD, a new threat to public health beyond coronary artery disease, involves aberrant vascular endothelial cell proliferation and angiogenesis [2, 4, 12]. Patients with PAD may develop MALE during the late stage of disease propagation, which markedly reduces blood flow to the extremities and progresses to the point of severe resting pain and even tissue loss [4, 12]. Although surgical or endovascular revascularization has been used for the treatment of PAD, some patients fail to tolerate these intricate and high-risk surgeries [4]. Notably, aging has been found to impair the angiogenic response to ischemic injuries, but the related molecular mechanisms remain largely unknown [13–15]. Herein, we, for the first time, showed that STAT3, a key player in cardioprotective ischemic conditioning [6], could be a biomarker predicting the development of MALE in patients and also a critical activator of age-dependent angiogenesis post limb ischemia.

Aging is not only a dominant risk factor for coronary artery diseases but also contributes to impaired collateral growth under ischemia by obliterating angiogenesis and endothelial function [14, 16]. Angiogenesis is both an essential adaptive response to physiological stress and an endogenous repair mechanism after ischemic injury [2, 10, 16]. The previous literature focuses on age-related endothelial dysfunction and the subsequent pathological remodeling of the microcirculation, which compromises tissue perfusion [16]. Through transplanting bone marrow stromal cells from young or old donors into the ischemic hind limbs of young or old rats restoring regional perfusion, Zhou documented a greater angiogenic potential in young donor cells than in old donor cells [17]. Kokubun et al. indicated that given low telomerase activity, increased ROS and DNA damage, under ischemic conditions collateral growth is impaired in aged animals [18]. Using superoxide-lowering therapy Fleenor et al. reported a reversal of aging related arterial dysfunction in mice [13]. Notably, VEGF remains a widely studied factor of angiogenic growth, and several phase II clinical trials are ongoing [19]. However, given the assumption that the lack of VEGF receptors in the muscle of patients with PAD is the main issue instead of the VEGF protein per se, currently, no trial has yielded optimal results in limb salvage [19]. Thus, searching for a novel target that regulates gene transcription to facilitate the growth of microcirculation may shed light on angiogenesis-based therapy for PAD.

STAT3, a pivotal transcription factor in cell proliferation and survival signaling, has been found to activate angiogenesis in endothelial cells in response to stresses [6, 9, 20]. Chen et al. indicated that VEGF stimulation of cultured ECs induced STAT3 phosphorylation by a VEGFR2- and Src-dependent mechanism [6]. Likewise, by analyzing human PAD muscle biopsies, Ganta et al. reported that VEGF_165_b prevents the activation of VEGFR1-STAT3 signaling by inhibiting angiogenesis and perfusion recovery in PAD muscle [10]. Their findings demonstrated that VEGFR1 activation and the resulting STAT3 activation also play a key role in improving perfusion recovery [20]. Tierney et al. identified an age predominate role of JAK-STAT3 signaling in inducing muscle stem cell homeostasis. Their findings highlighted that genetic and pharmacologic inhibition of STAT3 signaling improves the physiology of aged and dystrophic muscles. Additionally, by examining the impact of adipose-derived stromal cell treatment on aging hindlimb ischemia, Fan reported that adipose-derived stromal cells augmented inflammation-induced angiogenesis to yield proangiogenic/antiapoptotic effects via the VEGF/STAT3 pathway [21]. Likewise, miR-124-3p regulates angiogenesis in PAD by targeting STAT3 [22]. Taken together, the abovementioned findings support that STAT3 is involved in the angiogenetic process, but whether it also regulates the aging-related impairment of angiogenesis remains uncertain. In this study, we found that STAT3, beyond the traditional risk factors of PAD, could be a biomarker associated with the outcomes of limb salvage and also a critical regulator of age-dependent angiogenesis post limb ischemia. Furthermore, our immunofluorescence studies revealed the expression of MMP9 and VEGF in CD31 expressing endothelial cells. Furthermore, in aged mice subjected to PAD injury, the expression of VEGF and MMP9 in endothelial cells was lower than that of in young mice. These results demonstrated that STAT3 activation helps mediate VEGF induction and angiogenesis in endothelial cells.

Biomarkers are frequently used in clinical practices for diagnosis and risk stratification [23]. In patients with PAD, biomarkers facilitate physicians in identifying high-risk populations who may have poor outcomes in limb salvage [24]. Through a systemic review focusing on 21473 PAD patients, Kremers et al. found that high-sensitivity CRP levels had a 1.86 fold risk for MALEs [25]. However, other biomarkers, including fibrinogen, D-dimer, N-terminal pro-B-type natriuretic peptide and high-sensitivity cardiac troponin T, were only associated with the risk of mortality instead of the risk of MALE [25]. Thus, an optimal biomarker for determining the risk for progression and the response to therapy is lacking. Most importantly, the biomarker should pathologically explain its role in mitigating blood flow insufficiency in limb ischemia. Herein, among 216 patients receiving PTA for PAD, we observed that low expression of circulating STAT, especially in older patients, was significantly associated with the occurrence of MALEs. Additionally, in a rat model of hindlimb ischemia, aged mice had significantly low angiogenesis accompanied by low muscular expression of STAT3. Further, in aged HUVECs, proliferation, migration and tube formation were significantly impaired compared with young cells after hypoxia stimulation. Mechanistically, suppressing the expression of STAT3 causes HUVECs to lose their angiogenesis capability. Collectively, our findings pave the way toward pharmacological intervention targeting STAT3 activity to improve angiogenesis in patients with PAD.

There are some limitations of this study. First, given that aging is a complex process, a number of signal transduction pathways may be involved in aging-associated impairment of angiogenesis [20, 23]. Although comprehensive genetic profiling may provide clues regarding targets that have the potential to regulate the process of angiogenesis, as shown in our study, we found that STAT3 could sufficiently impact endothelial functions under hypoxia. Second, to establish an *in vitro* aged cellular model, previous studies have applied oxidative stresses, including hydrogen peroxide or high glucose, which is different from natural aging [26, 27]. Instead, we defined cells as less than 10 passages and above 30 passages as young and old cells, respectively, given that he doubling time o HUVEC cells were ~23.5 hours in the younger group (passage between 18 and 30) and ~100 hours in the older group (passage between 32 and 34) [28]. Previous reports also supported that aged cells increased β-galactosidase accumulation and high expression of senescence-associated genes, including p16, p21, and hTERT [11]. Thus, in our *in vitro* study, the aged HUVE cells were defined as passaged ≥30 presenting with aging characteristics, including a significant accumulation of β-galactosidase and high expressions of aging-associated genes. Finally, although proteins including VEGF and MMP9 may not be only expressed in endothelial cells but other muscle or inflammatory cells [29–31], the major cells responsible for angiogenic response to ischemia is endothelial cells [32] Thus, hereby we focused on the effect of aging on endothelial cells and their responses to hypoxia. Using co-staining with CD31 and MMP9 or CD31 and VEGF in the hindlimb muscles, we found that in both young and aged mice, expressions of MMP9 and VEGF were localized in CD31 expressing endothelial cells. However, we did not exclude the possibilities that those protein expressions could be secreted by surrounding cells other than endothelial cells.

Collectively, aging not only increases the prevalence of PAD but also obliterates vascular endothelial cell proliferation and angiogenesis. Mechanistically, we found that STAT3 could be a pivotal regulator in age-dependent angiogenesis post limb ischemia. Additionally, as a biomarker, it is highly associated with the development of MALE in patients with PAD. With the potential to be a therapeutic target, further studies are mandatory to focus on elucidating the usefulness of STAT3 in clinical applications.

## MATERIALS AND METHODS

### The surgery of limb ischemia in mice

17 Young (10-week-old) and aged (80–100-week-old) mice were randomly assigned to (1) control young group, (2) control aged group, (3) young hindlimb ischemia group, (4) aged hindlimb ischemia group. As described previously, a model of hindlimb ischemia were established in the left hind limbs of mice [12, 33]. Briefly, mice were anesthetized with an intraperitoneal injection of pentobarbital (80 mg/kg). The left femoral vein and artery were exposed through a transverse skin incision. The femoral vein and artery were ligated with 6–0 silk. The segment of vessels between the two ligations were excised. The mice of control group received sham surgery, in which the femoral artery and vein were exposed similar to the method above without ligation or excision, were performed on the contralateral leg. The detailed experimental design is shown in Supplementary Figure 5.

### LDI

The measurement of hindlimb blood flow were performed with a laser Doppler perfusion imaging analyzer (PeriScan PIM 3 Systems; Perimed AB, Sweden). Mice were measured before, immediately after surgery (Day 0), and Day 7, 14, 21, and 28. The digital color-coded images were analyzed to quantify blood flow in the region from the knee joint to the toe, and mean perfusion values were calculated. The laser Doppler perfusion imaging index was calculated as the ratio of perfusion in the ischemic versus the nonischemic hind limb.

### Calf muscle weight loss

After the end of experiment, calf muscles were harvested and weighed loss of mass in the ischemic muscle was expressed as the ischemic (left) calf muscle/nonischemic (right) calf muscle.

### Capillary density in ischemic limbs analysis

The calf muscles obtained from the control and the ischemic hind limbs were fixed in 4% paraformaldehyde and embedded in paraffin. The tissue sections were stained with the fluorescence-labeled antibody for CD31 (anti-human CD31, Alexa Fluor^®^ 594; BioLegend, San Diego, CA, USA). The nuclei were stained with 4’, 6-diamidino-2-phenylindole (DAPI; Abcam, Cambridge, MA, USA). The images were captured with a fluorescence microscope (Olympus BX51, Olympus Optical Co. Ltd, Tokyo, Japan). The Capillary density was counted in five random microscopic fields and analyzed by counting the mean area of CD31 expression.

### Histological analyses

Following 28 days of hindlimb ischemic surgery, the muscle of the ischemic hindlimb was excised and fixed in 4% paraformaldehyde and paraffin-embedded (Alfa Aesar, Lancashire, UK). For immunofluorescence double staining analyses, the muscle sections were stained for MMP9 (Arigo Biolaboratories) or VEGF (Thermo Fisher Scientific, MA, USA), followed by washing and stained with fluorescence-labeled antibody for CD31 (anti-human CD31, Alexa Fluor^®^ 594; BioLegend, San Diego, CA, USA). After washing, the specimens were stained with DAPI (Abcam, Cambridge, MA, USA) for visualization of nuclei. The images of the specimen were collected with fluorescence microscope (Olympus BX51, Olympus Optical Co. Ltd, Tokyo, Japan). Five different fields from each tissue preparation were randomly selected, and CD31 and MMP9 or VEGF double-positive cells were counted.

### Cell culture

Human umbilical vein endothelial cells (HUVECs) were purchased from ATCC (Manassas VA, USA) and cultured in M200 medium (Gibco, Thermo Fisher Scientific, MA, USA) supplemented with 10% fetal bovine serum, 100 units/ml penicillin, and 50 units/ml streptomycin (Invitrogen, Thermo Fisher Scientific, MA, USA). Cells were maintained in a humidified incubator of 5% CO_2_ at 37°C. The cells were cultured ≦10 passage and ≧30 passage as young and aged endothelial cells, respectively. In order to mimic endothelial cells under ischemic conditions as a model for PAD, HUVEC were subjected to hypoxia (0.1% oxygen; BioSpherix Medical, NY, USA).

### SA-β-galactosidase staining

Aged (≧30 passage) HUVEC were fixed in 4% paraformaldehyde and the senescent was verified by *β*-galactosidase staining using a kit in accordance with the manufacturer instructions [11]. In briefly, cells were incubated with β-galactosidase staining solution (X-gal; Sigma-Aldrich; Merck KGaA, Darmstadt, Germany) including 1 mg/ml 5-bromo-4-chloro-3-indolyl—D galactoside, 5 mM potassium ferrocyanide, 5 mM potassium ferricyanide, and 2 mM MgCl_2_ for 24 hr at 37°C. After stained, cells were observed under an inverted microscope (Leica Science Lab., Berlin, Germany). The percentage of SA-β-galactosidase positive cells were determined by counting the number of blue-stained cells. Three vision fields were randomly selected, and 100 cells were counted to calculate the senescence rate [senescence rate (%) = positive senescent cell/100 cells × 100%].

### RNA extraction and reverse transcription-quantitative polymerase chain reaction (RT-qPCR) analysis

Total RNA was isolated from HUVEC with Trizol (Ambion) following the manufacturer’s instructions. Single-stranded cDNA was synthesized using the reverse transcription PCR protocol of the first-strand cDNA synthesis kit (Invitrogen; Thermo Fisher Scientific, Inc.). RT-qPCR was performed on 7500 Fast Real-Time PCR system (Applied Biosystems, Foster City, CA, USA) with gene-specific primers using a OmicsGreen 5X Real-Time PCR MasterMix (Omics Bio). Primer sequences for detection of P16INK4a, P21, and telomerase reverse transcriptase (TERT) mRNA expression were as follows: P16INK4a-F: 5′-atatgccttcccccactacc-3′ and P16INK4a-R: 5′-cccctgagcttccctagttc-3′; P21-F: 5′-accgagacaccactggaggg-3′ and P21-R: 5′-cctgcctcctcccaactcatc-3′; hTERT-F: 5′-gccttcaagagccacgtc-3′ and hTERT-R: 5′-ccacgaactgtcgcatgt-3. Gene expression levels were calculated relative to the housekeeping gene GAPDH.

### Proliferation assay in HUVECs

HUVEC proliferation were measured with BrdU Cell Proliferation Assay Kit (Abcam, Cambridge, MA, USA) using in 96-well plates according to the manufacturer’s recommended protocol [34]. In brief, 2 × 10^5^ cells of HUVEC were seeded into wells allowed to grow for 24 hours at 37°C and then BrdU was added to the medium and the incubation was continued for an additional 24 hours under normoxia or hypoxia. After incubation, the cell proliferation was determined using a spectrophotometer (MULTISKAN. GO, Thermo Fisher Scientific, CA, USA) with a wavelength set at 450 nm.

### Migration assay

The 2 × 10^5^ of HUVEC were seeded in a culture-insert (ibidi culture-insert 2 well, ibidi GmbH, Martinsried, Germany). After cultured 24 hours, cells were removed the culture-insert and washed the cells with PBS to remove non-adherent cells [34]. The culture was continued with normoxia or hypoxia (0.1% oxygen; BioSpherix Medical) and was be set as 0 hours. The Images of cell migration were taken by light microscope (Olympus BX51, Olympus Optical Co. Ltd, Tokyo, Japan) at 0 and 6 hours. Cell migration distance was measured using the Image J software. Three independent experiments were performed.

### Tube formation assay

The ability of tube formation of HUVECs were evaluated by Matrigel assay (BD Biosciences, San Jose, CA, USA) as described previously study [33, 34]. Briefly, 2 × 10^5^of HUVEC were seeded on a 24-well plate coated with Matrigel basement membrane matrix (CORNING, Thermo Fisher Scientific, CA, USA). After culture, the tube formation was observed under the inverted microscope (Olympus BX51, Olympus Optical Co. Ltd, Tokyo, Japan) and the total length of the tubules per well in five rand selected fields (100×) were measured by ImageJ software (National Institutes of Health, Bethesda, MD, USA).

### STAT3 small interfering RNA (siSTAT3) transfection

The HUVEC were transfected with ON-TARGETplus SMARTPools targeting STAT3 (Dharmacon; L-003544-00-002) or scrambled siRNA in antibiotic/serum free medium using Dharma FECT 1 Transfection Reagent (Horizon Discovery, Cambridge, UK) according to the manufacturer’s instructions. The sequences of siRNA were as follows: siSTAT3-1: GAGAUUGACCAGCAGUAUA, siSTAT3-2: CAACAUGUCAUUUGCUGAA, siSTAT3-3: CCAACAAUCCCAAGAAUGU, and siSTAT3-4: CAACAGAUUGCCUGCAUUG. The silencing efficacy of siSTAT3 was confirmed at the RNA level by qPCR.

### Western blotting

The procedure of Western blot analysis was descried as previously [10, 13]. In brief, the equal amounts (50 μg/lane) were extracted from the calf muscle tissue or HUVEC and separated by SDS-PAGE using 10~15% acrylamide gradients. The membranes were incubated with antibodies against Phosphoinositide 3-kinases (PI3K), Phospho-PI3K (1:1000; Cell Signaling, Danvers, MA, USA), AKT, Phospho-AKT (1:1000, Invitrogen, Thermo Fisher Scientific, MA, USA), STAT3 (1:1000; Abcam, Cambridge, MA, USA), Phospho-STAT3(Tyr705) (1:1000; Cell Signaling, Danvers, MA, USA), Matrix metallopeptidase 9 (MMP9) (1:1000, Arigo Biolaboratories, Taiwan), vascular endothelial growth factor (VEGF) (1:1000, Proteintech, Rosemont, IL, USA), VEGFR2 (1:1000) and Phospho-VEGFR2 (1:5000 Abcam, Cambridge, MA, USA). Signals were detected with HRP-conjugated goat anti-mouse or goat anti-rabbit IgG (Jackson Immuno Research Laboratories Inc., West Grove, PA, USA). Immunoreactive bands were detected by enhanced chemiluminescence (ECL; Thermo Fisher Scientific, CA, USA) and then were exposed to ECL-Western blotting system (AVEGENE CHEMX 400). The intensity of the protein band was quantified by Image J software (Bethesda, NIH, MD, USA) and the results are expressed as normalized ratio to housekeeping gene GAPDH.

### Patients and clinical study design

We prospectively included patients preparing for percutaneous transluminal angioplasty (PTA) for PAD in Chi-Mei Medical Center. Before interventions, the sera and clinical information were collected. Blood pressures and heart rates were measured before PTA. Patients were divided as old (≥65 y/o) and young (<65 y/o) populations. Patients who were lost follow-up, preparing for amputation, in a status of active infection or with an expected life span less than one year were excluded. The end-point of major adverse limb events (MALE) was defined as reintervention on the index arterial segment or amputation of the index limb. The median follow-up duration were 20 months (interquartile range, IQR: 12–38 months). The study was conducted in strict accordance with the Declaration of Helsinki on Biomedical Research involving human subjects and was approved by the local ethics committee (IRB: 10307-003). Also, this study is registered in Clinical Trials (protocol ID: CMMC10705-003).

### Statistical analysis

The chi-squared tests were used to compare differences in age and comorbidity frequencies between PAD patients with and without MALEs. After testing for normality, continuous variables were compared between young and aged PAD patients using the Mann-Whitney *U* test. The Kaplan-Meier method was used to plot MALEs, and group differences were compared via the log-rank test. The hazard ratio (HR) of MACCEs between cancer patients with and without diabetes was estimated using the Cox proportional hazard regression model adjusted for the potential confounding factors age and comorbidities. A two-tailed *P* value < 0.05 was considered statistically significant for all of the tests. All of the analyses were conducted using SAS software version 9.4 (SAS Institute, Cary, NC, USA). Kaplan-Meier curves were plotted using STATA (version 12; Stata Corp., College Station, TX, USA).

### Availability of data and materials

The data is available upon the reasonable request to the corresponding author.

## Supplementary Materials

Supplementary Figures

Supplementary Table 1
