# Supplementary material for: Effects of STAT3 on aging-dependent neovascularization impairment following limb ischemia: from bedside to bench
Source: Aging (Albany NY). 2022 Jun 13;14(11):4897–913. doi: 10.18632/aging.204122 (PMC9217700; doi:10.18632/aging.204122)
Supplement: Supplementary Figures [file aging-14-204122-s001.pdf]

SUPPLEMENTARY FIGURES

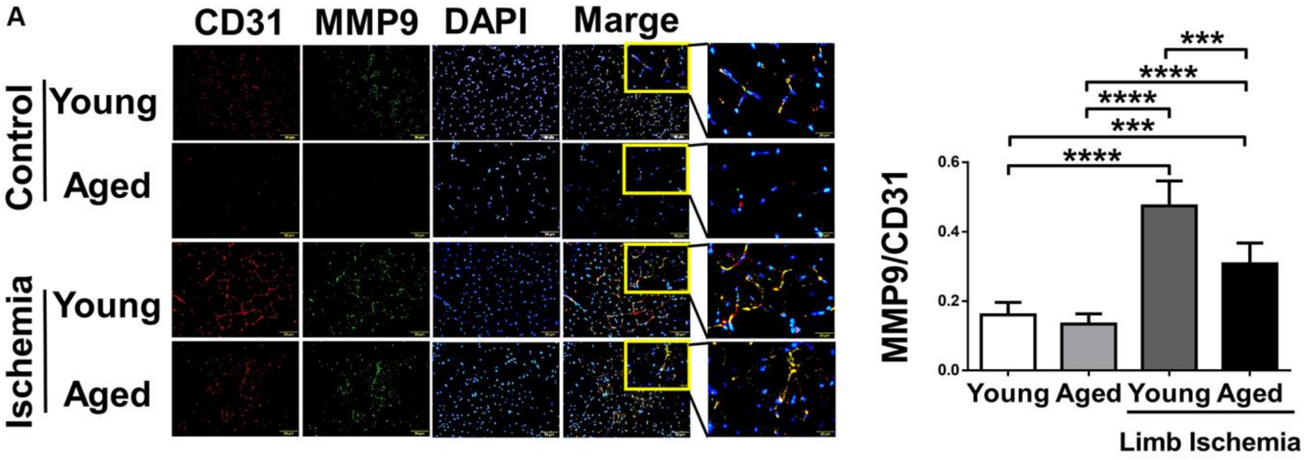

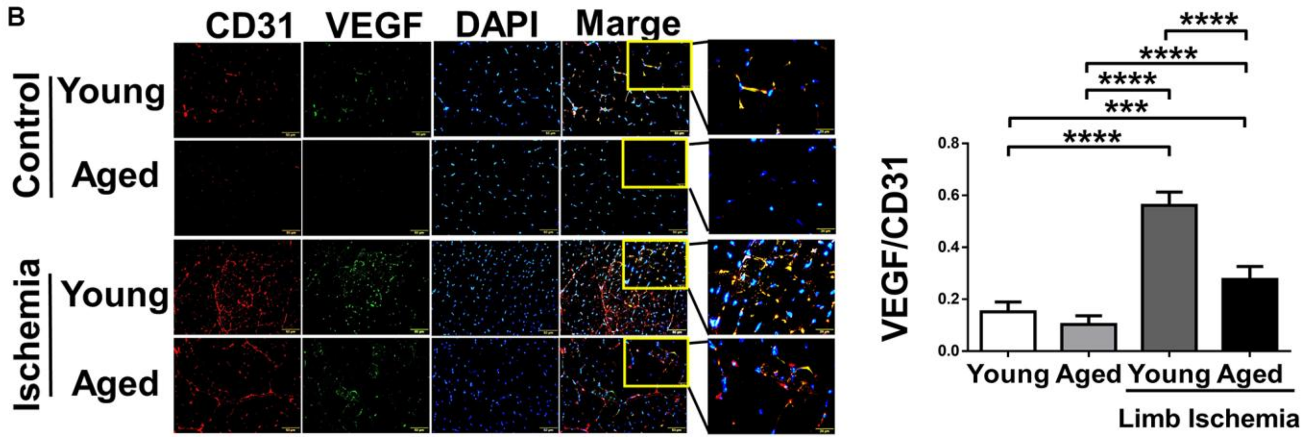

Supplementary Figure 1. Double immunostaining with endothelial marker CD31 and VEGF or MMP9 in young and aged mice post hindlimb ischemia surgery. Representative images and quantification of (**A**) VEGF (green) and (**B**) MMP9 (green) expressions in endothelial cells (red; CD 31 positive). Cell nuclei were stained with DAPI (blue). Scale bar: 50 μm. $^{***}P < 0.001$ and $^{****}P < 0.0001$ compared with the indicated groups.

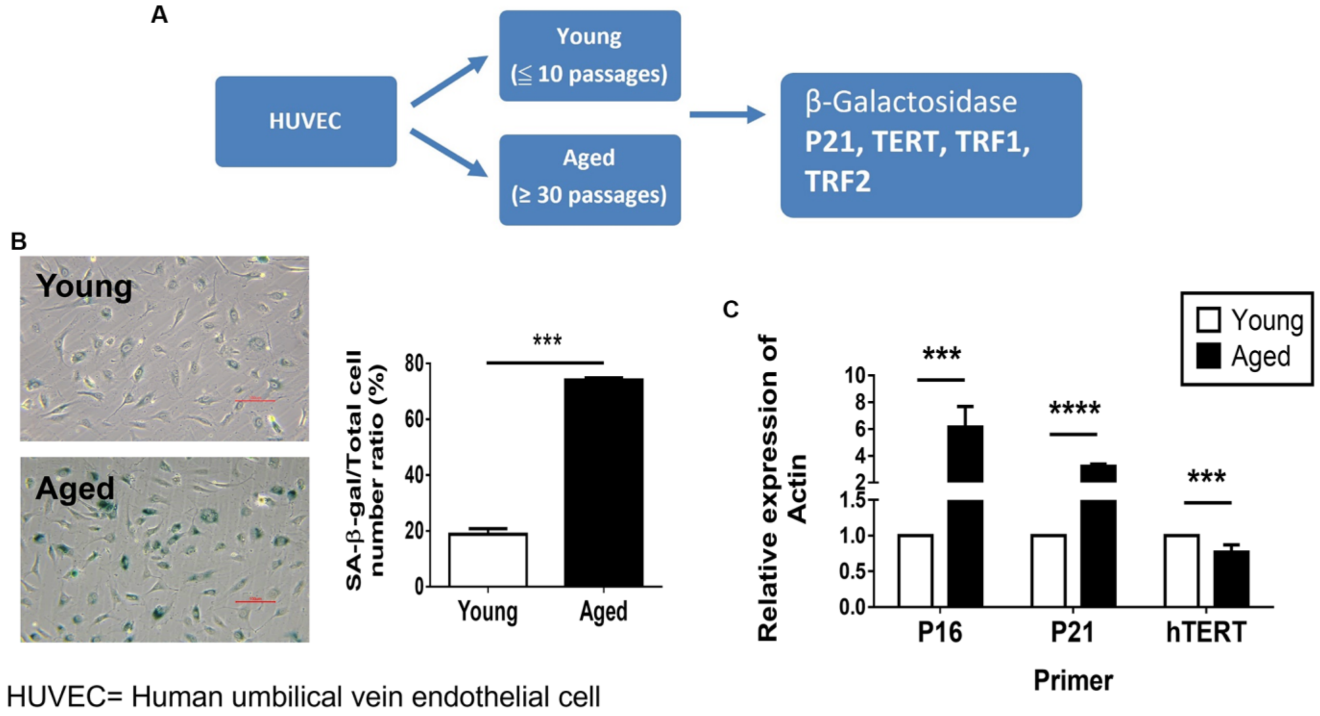

Supplementary Figure 2. The evaluation of aging in HUVECs. (**A**) Definitions of young (≤10 passages) and aged (≥30 passages) HUVECs. (**B**) Representative images and quantification of β-galactosidase staining in young and aged HUVECs. (**C**) Quantification PCR of P16INK4a, P21, and hTERT mRNA. $^{***}P < 0.001$ and $^{****}P < 0.0001$ compared with the indicated groups ($N = 3–6$).

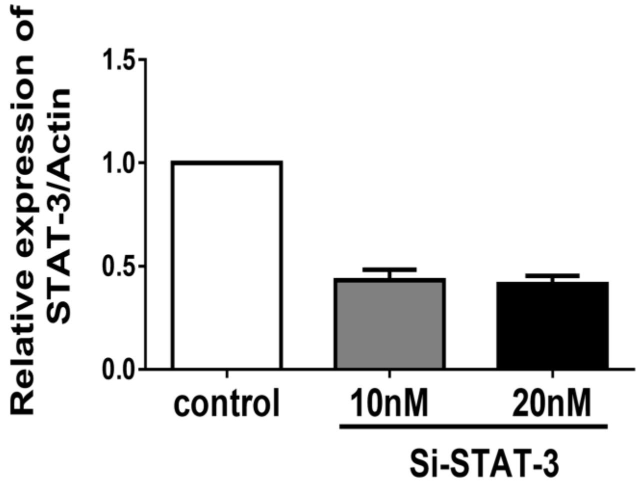

Supplementary Figure 3. The level of STAT3 in HUVECs transfected with STAT3 small interfering RNA (siSTAT3). HUVECs were transfected with siSTAT3 for 48 hours, and STAT3 mRNA expression was measured by qPCR.

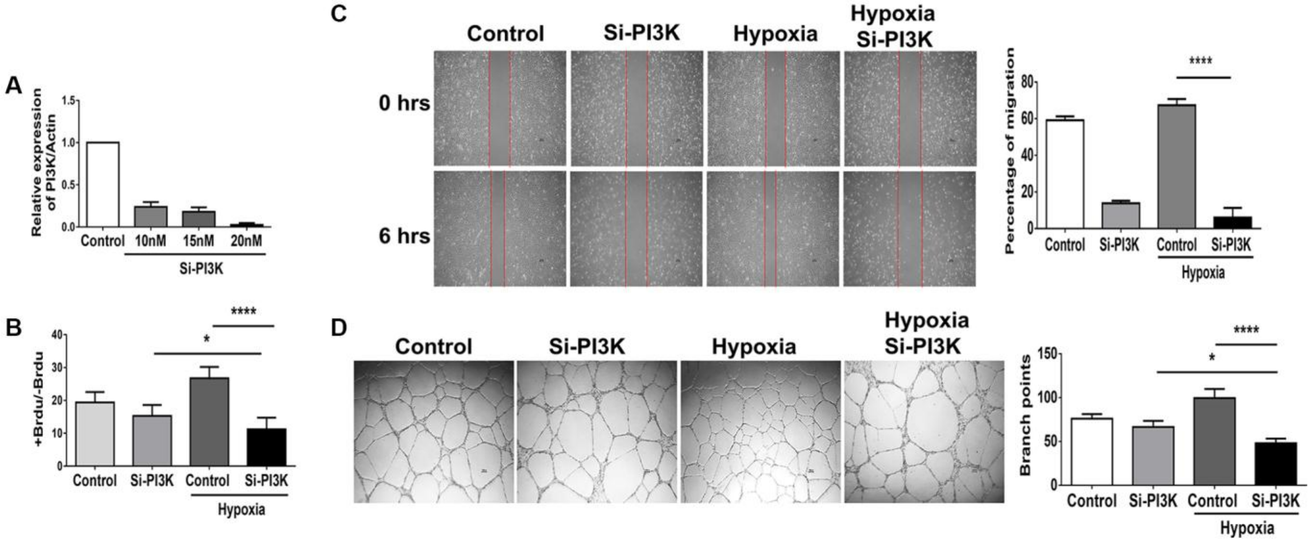

Supplementary Figure 4. Knocking down PI3K suppresses proliferation, migration and tube formation in human umbilical vein endothelial cells (HUVECs). (**A**) HUVECs were transfected with PI3K small interfering RNA (si-PI3K) for 48 hours, and PI3K mRNA expression was measured by qPCR. (**B**) Cell proliferation, evaluated by measuring BrdU incorporation into cells, (**C**) cell migration after 24 hours, and (**D**) tube formation, evaluated by the branched points, in HUVECs treated with scramble or si-PI3K under normoxia or hypoxia for 24 hours. The experiment was repeated in triplicate, $^{*}P < 0.05$ and $^{****}P < 0.0001$.

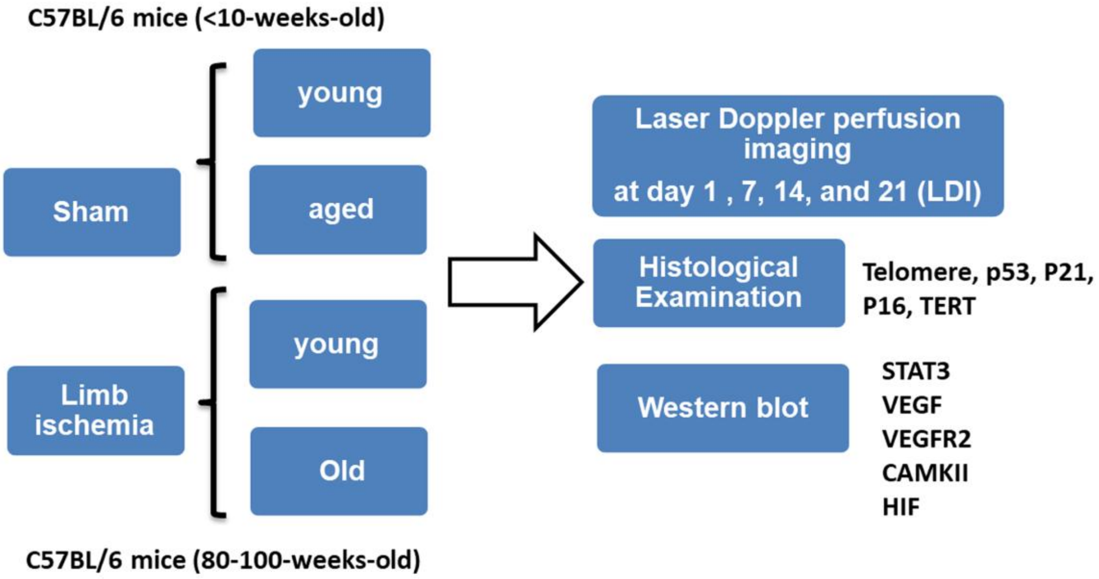

Supplementary Figure 5. The groups in the animal study. The study design for investigating the effects of angiogenesis in young (<10-week-old) and aged mice (80–100 week-old) with hindlimb ischemia. The images of laser Doppler perfusion flow and histological and angiogenesis-associated proteins were measured at the end of the experiment.
