# Supplementary material for: Effects of STAT3 on aging-dependent neovascularization impairment following limb ischemia: from bedside to bench
Source: Aging (Albany NY). 2022 Jun 13;14(11):4897–913. doi: 10.18632/aging.204122 (PMC9217700; doi:10.18632/aging.204122)
Supplement: Supplementary Table 1 [file aging-14-204122-s002.pdf]

SUPPLEMENTARY TABLE

Supplementary Table 1. The baseline characteristics of patients with peripheral arterial disease (PAD), divided by the development of major adverse limb events (MALEs) (*N* = 216).

Parameters	MALE (−) (*N* = 142)	MALE (+) (*N* = 74)	*P* value
Age (y/o)	62.5 ± 18.1	70.1 ± 11.5	0.05
Male gender, *n* (%)	92 (64.7)	47 (63.5)	0.88
BMI (kg/m2)	24.7 ± 4.4	24.6 ± 4.7	0.898
Heart rate (bpm)	81.7 ± 18.1	81 ± 14.9	0.766
SBP (mmHg)	151.7 ± 30.2	155.47 ± 27.1	0.375
DBP (mmHg)	77.2 ± 15.3	77.6 ± 16.2	0.855
Smoking, *n* (%)	93 (65.5)	44 (59.4)	0.43
DM, *n* (%)	82 (57.7)	48 (64.8)	0.14
HTN, *n* (%)	94 (66.1)	54 (72.9)	0.44
CAD, *n* (%)	57 (40.1)	39 (52.7)	0.08
HF, *n* (%)	13 (9.1)	14 (18.9)	0.05
Hyperlipidemia, *n* (%)	66 (46.4)	22 (29.7)	0.21
Previous stroke, *n* (%)	26 (18.3)	5 (6.7)	0.024
Cancer, *n* (%)	13 (9.2)	3 (4.1)	0.27
CKD (including H/D), *n* (%)	26 (18.3)	24 (32.4)	0.039
eGFR (ml/min/1.73m^2)	60.6 ± 33.1	51.7 ± 32.2	0.077
ALT (mg/dl)	23.2 ± 2.1	35.9 ± 4.5	0.82
Total Cholesterol (mg/dl)	178.1 ± 34.3	185.1 ± 59.4	0.92
LDL (mg/dl)	83.8 ± 14.6	98.8 ± 18.9	0.7
Triglyceride (mg/dl)	170.5 ± 191	183.5 ± 38.4	0.78
Circulating STAT3 (ng/ml)	17.2 ± 6.2	11.1 ± 5.9	0.001

Data are *n* (%) or mean ± standard error; $P < 0.05$ as significance. Abbreviations: BMI: body mass index; SBP: systolic blood pressure; DBP: diastolic blood pressure; CAD: coronary artery disease; CKD: chronic kidney disease; eGFR: estimated Glomerular filtration rate; ALT: Alanine aminotransferase; LDL: low-density lipoprotein; STAT3: Signal transducer and activator of transcription 3; MALE: major adverse limb events.
